# Increased utilisation of PEPFAR-supported laboratory services by non-HIV patients in Tanzania

**DOI:** 10.4102/ajlm.v5i1.318

**Published:** 2016-02-16

**Authors:** Margaret L. McNairy, Charon Gwynn, Miriam Rabkin, Gretchen Antelman, Yingfeng Wu, Bereket Alemayehu, Travis Lim, Rubina Imtiaz, Fausta Mosha, Michael Mwasekaga, Asha A. Othman, Jessica Justman

**Affiliations:** 1 ICAP, Columbia University, New York, New York, United States; 2 Weill Cornell Medical College, New York, New York, United States; 3 Centers for Disease Control and Prevention, Atlanta, Georgia, United States; 4 Ministry of Health and Social Welfare, Dar es Salaam, Republic of Tanzania; 5 Centers for Disease Control and Prevention, Dar es Salaam, Republic of Tanzania; 6 Ministry of Health, Zanzibar, Republic of Tanzania

## Abstract

**Background:**

It is unknown to what extent the non-HIV population utilises laboratories supported by the President’s Emergency Plan for AIDS Relief (PEPFAR).

**Objectives:**

We aimed to describe the number and proportion of laboratory tests performed in 2009 and 2011 for patients referred from HIV and non-HIV services (NHSs) in a convenience sample collected from 127 laboratories supported by PEPFAR in Tanzania. We then compared changes in the proportions of tests performed for patients referred from NHSs in 2009 vs 2011.

**Methods:**

Haematology, chemistry, tuberculosis and syphilis test data were collected from available laboratory registers. Referral sources, including HIV services, NHSs, or lack of a documented referral source, were recorded. A generalised linear mixed model reported the odds that a test was from a NHS.

**Results:**

A total of 94 132 tests from 94 laboratories in 2009 and 157 343 tests from 101 laboratories in 2011 were recorded. Half of all tests lacked a documented referral source. Tests from NHSs constituted 42% (66 084) of all tests in 2011, compared with 31% (29 181) in 2009. A test in 2011 was twice as likely to have been referred from a NHS as in 2009 (adjusted odds ratio: 2.0 [95% confidence interval: 2.0–2.1]).

**Conclusion:**

Between 2009 and 2011, the number and proportion of tests from NHSs increased across all types of test. This finding may reflect increased documentation of NHS referrals or that the laboratory scale-up originally intended to service the HIV-positive population in Tanzania may be associated with a ‘spillover effect’ amongst the general population.

## Introduction

Investment in strengthening laboratory systems in resource-poor countries is critical to meet health needs across major diseases such as HIV/AIDS and to meet the United Nations Millennium Development Goals.^1^ In the past decade, the US government has invested over $15 billion in HIV prevention, care and treatment in low- and middle-income countries via the President’s Emergency Plan for AIDS Relief (PEPFAR).^2^ This support has included a wide range of activities aimed at strengthening health services, including laboratories, to provide services for persons living with HIV (PLWH). Although the positive impact of these targeted health services on PLWH is undeniable, the effect of HIV service scale-up on broader health systems, including services for patients without HIV, has been debated.^3,4,5,6,7^

Since 2006, PEPFAR has provided over $440 million to strengthen laboratory systems through improved infrastructure and equipment, human resources and training, quality improvement, and technical assistance.^8^ This investment has expanded laboratory services such as diagnostic and monitoring tests for PLWH. Because these laboratory investments support health facilities serving a broad population of patients, not just PLWH, it is plausible that they may have affected, or in the future could affect, the coverage and quality of laboratory services used by the general population – that is, individuals with no known HIV infection.^9^ To our knowledge, no studies have explored this question yet.

In an effort to describe PEPFAR’s investment in laboratory services for the general population, we analysed routinely collected programmatic data from selected public laboratories in Tanzania. Specifically, we selected a convenience sample of PEPFAR-supported laboratories in Tanzania, which are supported through ICAP at Columbia University.^10^ In these laboratories, the only information distinguishing the HIV status of the patient from whom the test was collected was the test’s referral source; that is, an HIV service or a non-HIV service (NHS) (e.g. general medical or outpatient services). Although referral source is not a definitive diagnosis of HIV status, it was the only routinely recorded information available as a proxy for HIV status. Our primary objective was to describe the number and proportion of selected core laboratory tests performed for patients referred from the respective services in 2011. A secondary objective was to compare changes in proportions of tests performed for patients referred from NHSs in 2009 and 2011.

## Research method and design

### Ethical considerations

This study was approved by the Columbia University Medical Center Institutional Review Board, the US Centers for Disease Control and Prevention, the Tanzania National Institute for Medical Research and the Zanzibar Medical Research and Ethics Committee.

### Study population

We conducted a retrospective cross-sectional analysis of laboratory tests from 2009 and 2011 in a convenience sample of PEPFAR-supported public laboratories in Tanzania. All laboratories received PEPFAR support from ICAP at Columbia University. Laboratories that were included were all categorised as public sector, offered integrated laboratory services for all laboratory samples (i.e. using the same staff and equipment for HIV and non-HIV patients), performed at least haematology testing over the study period, and had available laboratory register data on preliminary assessment. Data abstracted from laboratory testing registers did not include patient-identifying information.

### Definitions of laboratory tests and outcomes

A laboratory test was defined as the presence of a documented haematology, chemistry, tuberculosis or syphilis test result in a laboratory register located at the laboratory facility. A haematology test result was defined as any automated or manual test for haemoglobin or a complete blood count (e.g. Celldyne 1800, Coulter). A chemistry test result was defined as creatinine or liver function tests (alanine aminotransferase, aspartate aminotransferase or alkaline phosphatase) or other blood chemistry panel results from an automated machine (e.g. Humastar 80, Hitachi, Reflotron). A tuberculosis test included a microscopy smear or culture. A syphilis test result was defined as a test from a venereal disease research laboratory or a rapid plasma reagin or antibody test. On-site registers were used to classify samples as from HIV services, a NHS, or an unknown referral source (i.e. did not have a documented referral source).

The primary outcome of this study was the proportion of laboratory tests with documented NHS referral sources amongst all tests with a referral source (either HIV or NHS referral). Other outcomes included the proportion of laboratory tests performed with documented NHS referral sources amongst all tests, including tests with and without referral sources.

### Site-level variables

Programmatic information was used to provide contextual information about included laboratories. Routinely collected quarterly monitoring and evaluation data from the co-located HIV care and treatment facilities were used to quantify the number of years each facility had provided HIV care services and the number of HIV-positive patients enrolled in the HIV care service. Information from facility-based surveys completed in 2009 and 2011 at laboratories included the location type (urban vs rural) and type of facility (primary, secondary or tertiary); the 2011 survey also described the number of trained laboratory personnel working in each laboratory.

### Data collection

Between March and July of 2013, study staff extracted de-identified laboratory data from on-site hard-copy registers at included laboratories. Study staff met briefly with laboratory personnel to assess the availability of laboratory registers for each of the aforementioned tests. Study staff reviewed the available laboratory registers to tally the number of each type of test conducted per month. Totals were aggregated by the type of referral source. If an HIV clinic was indicated as a referral source, the test was categorised as coming from an HIV service. If another clinic or unit within the facility was documented as the source in the register, the test was categorised as coming from a NHS. If no source was documented for the patient, the test was categorised as coming from an unknown referral source.

### Statistical analysis

Proportions of tests conducted amongst all the laboratories were calculated for specimens referred from HIV services, NHSs and those with an unknown referral source. Proportions were calculated by year and by test type. A generalised linear mixed model was constructed to predict the odds that a laboratory test was referred from a NHS, taking into account intrafacility correlations. We used a generalised linear mixed model without confounders to account for intrafacility correlation, and an adjusted generalised linear mixed model that controlled for key facility-level variables including year, facility location and total volume of tests performed at each facility as fixed effects, with the laboratory treated as the random effect. Key contextual variables hypothesised to affect the proportion of tests from an HIV service compared with from a NHS, such as location (rural vs. urban), region, facility type and service size (e.g. number of patients enrolled in HIV care) were assessed individually to determine an unadjusted odds ratio. Candidate confounders (*P* < 0.25 when unadjusted) were entered and examined in generalised linear mixed models, but only the significant variables (*P* < 0.05) were kept in the final models for the purposes of calculating the adjusted odds ratios. All analyses were conducted using SAS version 9.3 (SAS Institute, Cary, North Carolina, United States).

## Results

**FIGURE 1:**
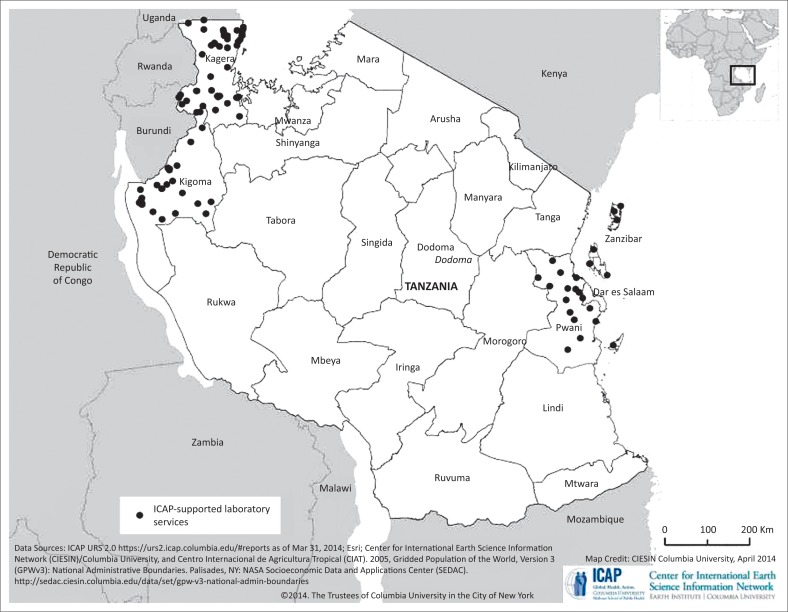
Location of laboratories in Tanzania receiving PEPFAR support from ICAP during 2009 and 2011 (*N* = 94 laboratories in 2009 and 101 in 2011).

Amongst the 127 PEPFAR-supported laboratories in Tanzania during the study period, 94 laboratories had testing data available from registers in 2009 and 101 laboratories had testing data available from registers in 2011 (Figure 1). A total of 93 laboratories had testing data for both 2009 and 2011. When the analysis was restricted to laboratories whose registers included tests with a referral source, a total of 51 in 2009 and and 61 laboratories in 2011 remained in the sample.

### Characteristics of laboratory facilities

The majority of laboratories were located in an urban area (Table 1). In 2009, there were 59 (63%) primary level laboratories, whereas in 2011 there were 66 (65%). In 2009, 70% of laboratories had been providing HIV care for up to one year, compared with 2% in 2011; in 2011, 67% had been providing HIV care for two to three years and 31% had been providing HIV care for at least four years. The median number of PLWH enrolled in care at PEPFAR-supported HIV facilities increased from 139 in 2009 to 269 in 2011. Data were not available on the median number of laboratory technicians in 2009, but 40% of laboratories had one technician in 2011, 32% of laboratories had two or three technicians in that year and 29% had at least four technicians. The median number of laboratory tests documented in available on-site registers was 415 tests (interquartile range [IQR]: 108–1211) in 2009 and 652 tests (IQR: 217–2034) in 2011. The number and proportion of laboratories conducting more than 1500 tests per year increased from 18 (19%) in 2009 to 28 (28%) in 2011.

**TABLE 1:** Characteristics of PEPFAR-supported laboratories in Tanzania for which data were provided for 2009 and 2011.

Laboratory characteristics	2009		2011	
	
	*N*	%	*N*	%
**All laboratories**	94	100	101	100
**Location type**				
Urban	49	52	51	50
Rural	34	36	37	37
Missing data	11	12	13	13
**Type of facility**				
Primary	59	63	66	65
Secondary	34	36	34	34
Tertiary	1	1	1.0	1
**Region**				
Kagera	41	44	45	45
Kigoma	28	30	30	30
Pwani	18	19	19	19
Zanzibar	7	7	7	7
**Median number of years of HIV care services**				
Median (IQR)	1 (1, 2)		3 (2, 4)	
≤ 1 year	66	70	2	2
2–3 years	16	17	68	67
≥ 4 years	12	13	31	31
**Number of HIV-positive patients enrolled in care**†				
Median (IQR)	139 (34, 558)		269 (82, 947)	
< 100	34	36	25	25
100–499	25	27	31	31
≥ 500	22	23	32	32
Unknown	13	14	13	13
**Number of laboratory technicians**				
Median (IQR)	n/a		2 (1,4)	
1	n/a		40	40
2–3	n/a		32	32
≥ 4	n/a		29	29
**Test type**				
Haematology	60	64	93	92
Chemistry	12	13	23	23
Tuberculosis microscopy	80	85	89	88
Syphilis	30	32	46	46
**Total number of tests conducted per laboratory**				
Median (IQR)	415 (108, 1211)		652 (217, 2034)	
≤ 500	51	54	37	37
501–1500	25	27	36	36
> 1500	18	19	28	28

† Data collected from a sample of the 127 PEPFAR-supported laboratories in Tanzania. IQR, interquartile range.

The completeness of available data at laboratories varied according to the type of test and over time. The proportion of laboratories providing any data on haematology tests increased from 64% (60/94) in 2009 to 92% (93/101) in 2011 (*P* < 0.001) (Table 1). The proportion of laboratories providing any data on other tests increased measurably but not significantly from 2009 to 2011: 13% (12/94) vs. 23% (23/101) for chemistry tests (*P* = 0.07), 85% (80/94) vs. 88% (89/101) for tuberculosis tests (*P* = 0.53), and 32% (30/94) vs. 46% (46/101) for syphilis tests (*P* = 0.05). Of the 94 laboratories providing data in 2009, 61% (57/94) provided data for 12 months of the year compared with 75% (75/101) in 2011 (data not shown in table 1).

### Characteristics of laboratory tests

**FIGURE 2:**
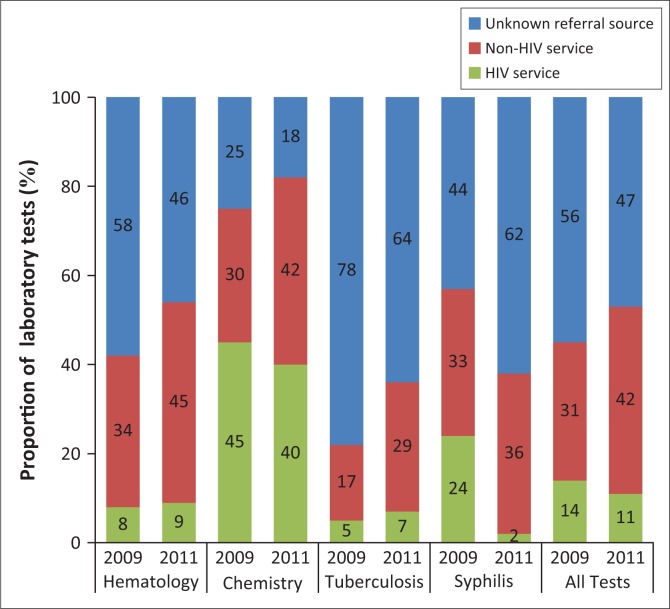
Referral sources for laboratory tests performed by PEPFAR-supported laboratories in Tanzania, 2009 and 2011. The graph depicts the overall proportion of laboratory tests performed for patients from different types of referral source (94 laboratories in 2009; 101 laboratories in 2011).

The total number of tests recorded increased from 94 132 in 94 laboratories in 2009 to 157 343 in 101 laboratories in 2011 (Table 2). The proportion of all tests performed for patients referred from a NHS increased from 31% (29 181) in 2009 to 42% (66 084) in 2011 (Figure 2). In both years, less than one-fifth of all tests were documented as being referred from HIV services: 14% (13 178) in 2009 and 11% (17 308) in 2011. Approximately half of all tests lacked a documented referral source: 56% (52 714) in 2009 and 47% (73 951) in 2011.

**TABLE 2:** Tests conducted by PEPFAR-supported laboratories in 2009 and 2011 by type of test

Year	Type of test No. (percentage)				All tests

	Haematology	Chemistry	Tuberculosis	Syphilis	
2009†	54 449 (58%)	12 607 (13%)	17 498 (19%)	9528 (10%)	94 132
2011‡	104 693 (67%)	17 680 (11%)	21 550 (14%)	13 420 (9%)	157 343

† Includes test data from 94 laboratories;

‡ Includes test data from 101 laboratories.

Haematology tests constituted the majority of all tests documented in the two study periods, accounting for 58% (54 499) of all tests in 2009 and 67% (104 693) of tests in 2011 (Table 2; Figure 2). The proportion of haematology tests performed for patients referred from a NHS increased from 34% in 2009 to 45% in 2011. Less than 10% of haematology tests in either year were performed for patients with a documented referral from HIV services (8% in 2009 and 9% in 2011). In contrast, chemistry tests represented a much smaller proportion of the total number of tests: 13% (12 607) in 2009 and 11% (17 680) in 2011. However, the proportion of chemistry tests performed for patients referred from HIV services was much larger than any other test type (45% in 2009 and 40% in 2011). The vast majority of all tuberculosis tests in 2009 and 2011 had an unknown referral source: 78% (13 648) in 2009 and 64% (13 792) in 2011. Syphilis testing increased from 9528 tests in 2009 to 13 420 tests in 2011. The proportion of syphilis tests recorded for patients referred from HIV services decreased from 24% (2287) in 2009 to 2% (268) in 2011 and was accompanied by a large increase in the proportion of tests performed for patients with an unknown referral source: from 44% (4192) in 2009 to 62% (8320) in 2011.

**FIGURE 3:**
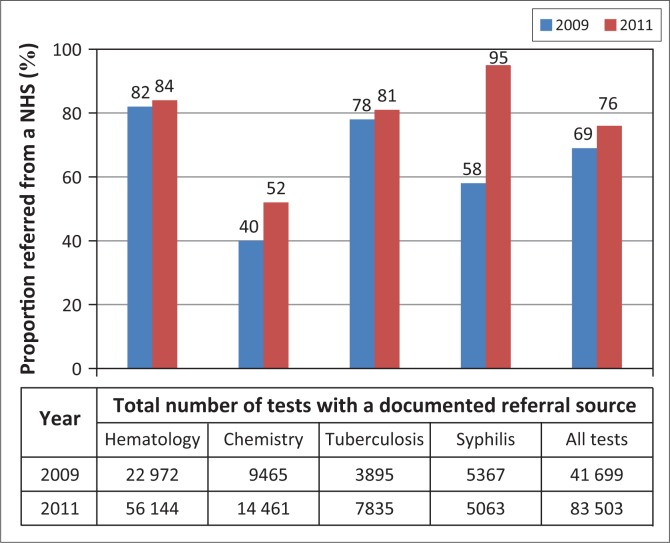
Proportion of tests referred from non-HIV services in 2009 and 2011 from amongst tests with a documented referral source. After excluding tests without a documented referral source, a total of 51 laboratories in 2009 and 61 laboratories in 2011 remained for the analysis. The total number of tests for which any referral status was available is shown for each year below the graph for each test category.

When analyses were restricted only to tests with a documented referral source, the sample of laboratories decreased from 94 to 51 in 2009 and from 101 to 61 in 2011. Amongst this sample, the proportion of all laboratory tests performed for patients referred from NHSs increased from 69% (28 722) in 2009 to 76% (63 462) in 2011 (Figure 3). The proportion of haematology tests performed for patients referred from NHSs increased modestly from 82% (18 722) in 2009 to 84% (46 936) in 2011, yet dramatically in net number. The proportion of chemistry tests performed for patients referred from NHSs increased from 40% (3795) in 2009 to 52% (7461) in 2011, and the proportion of tuberculosis diagnostic tests increased from 78% (3046) to 81% (6323) during this same period. The proportion of syphilis tests performed for patients referred from NHSs increased the most: from 58% (3107) in 2009 to 95% (4810) in 2011.

For all laboratory tests, tests were approximately twice as likely to be referred from NHSs in 2011 compared with 2009, based on the unadjusted odds ratio of 1.9 (95% confidence interval [CI]: 1.8–1.9) (Table 3). A similar odds ratio was estimated after adjusting for year, location and number of tests conducted (adjusted odds ratio: 2.0 [95% CI: 2.0–2.1]). When stratified by test type, we found that amongst all types of test, chemistry and syphilis tests were the most likely to have been referred from NHSs in 2011 compared with 2009 (adjusted odds ratios: 1.9 [95% CI: 1.7–2.0] for chemistry; 13.0 [95% CI: 11.0–17.0) for syphilis]). Haematology tests performed in urban laboratories were more likely to be referred from NHSs than haematology tests from rural laboratories (adjusted odds ratio: 6.8 [95% CI: 1.2–40.0]). Samples that came from laboratories that conducted fewer tests (≤ 500 and 501–1500 per year) were more than twice as likely to be referred from NHSs compared with those from laboratories that conducted a larger number of tests (> 1500) per year (adjusted odds ratios: 2.5 [95% CI: 1.9–3.3] for ≤ 500 tests and 2.6 [95% CI: 2.3–2.9] for 501–1500 tests).

**TABLE 3:** Odds ratios of a laboratory test being performed for a patient referred from non HIV-services, 2009 and 2011.†,‡

Variable	Haematology		Chemistry		Tuberculosis		Syphilis		All tests	
				
	Unadjusted	Adjusted§	Unadjusted	Adjusted§	Unadjusted	Adjusted§	Unadjusted	Adjusted§	Unadjusted	Adjusted§
**Year**										
2009	1	1	1	1	1	1	1	1	1	1
2011	0.65 (0.6, 0.7)	0.75 (0.7,0.8)	1.9 (1.7, 2.0)	1.9 (1.7,2.0)	0.5 (0.4,0.6)	0.6 (0.5,0.7)	13.0 (11.0, 17.0)	13.0 (11.0,17.0)	1.9 (1.8, 1.9)	2.0 (2.0, 2.1)
**Location**										
Rural	1	1	1	-	1	-	1	-	1	-
Urban	4.8 (0.9, 26.0)	6.8 (1.2, 40.0)	0.1 (0.0,10.0)	-	1.4 (0.4, 5.2)	-	0.17 (0.0, 2.6)	-	1.0 (0.4, 2.4)	-
**Total number of tests**										
≤ 500	7.2(3.9, 13.0)	4.3 (2.0., 3.8)	-	-	4.2(2.5,7.2)	2.5 (1.4, 4.5)	15(1.1, 210.0)	-	0.95 (0.7, 1.2)	2.5 (1.9, 3.3)
501–1500	18.0 (12.0, 27.0)	14.0 (9.0, 21.0)	0.07 (0.0, 4.0)	-	3.6(1.0.9, 13.0)	3.3 (0.9, 13)	3.2(0.3, 38.0)	-	1.3 (1.2, 1.5)	2.6 (2.3, 2.9)
> 1500	1	1	1	-	1	1	1	-	1	1

† Compared with tests performed for a patient referred from an HIV service. Excludes tests with unknown referral source; includes 51 laboratories in 2009 and 61 laboratories in 2011;

‡ We were unable to examine the effect of facility type (primary, secondary, tertiary) or region owing to small sample sizes in the variable categories. As a result, they were not included in the final models. Empty cells indicate that the regression model did not converge and no estimate was calculated;

¶ Unadjusted odds ratios account for intrafacility correlation;

§ Adjusted for intrafacility correlation and facility-level characteristics including year, location and total number of tests performed at each facility.

## Discussion

We investigated the potential impact of PEPFAR-supported laboratory scale-up on the general (non-HIV) patient population in a country in sub-Saharan Africa. The results describe the number of laboratory tests performed in 2009 and 2011 in a convenience sample of PEPFAR-supported laboratories in Tanzania and the proportion of tests performed for patients referred from HIV services, NHSs and unknown sources. A key finding in this analysis is the substantial increase in the proportion of all tests referred from NHSs from 2009 to 2011 – both when including all laboratory tests and when including only tests with known referral sources.

There was considerable variation in the number of tests performed by each facility (IQR: 108–1211 tests in 2009 and 217–2034 in 2011). Also of note was the substantial variation in testing volume across different types of laboratory tests. Haematologic tests were the most common type and are the laboratory cornerstone for antenatal care, malaria diagnosis and treatment, routine outpatient diagnostics for infectious diseases, and HIV care. Syphilis tests were the least common test; however, the volume of syphilis tests increased substantially from 2009 to 2011, reflecting in part a Tanzania Ministry of Health recommendation for rapid tests kits (SD Bioline), which enabled routine point-of-care syphilis screening to become more feasible, as opposed to rapid plasma reagin, which require cold-chain analysis and trained laboratory staff.

Chemistry tests were performed most often for patients referred from HIV services, which may reflect the clinical practice of assessing renal function amongst HIV patients before and during antiretroviral therapy.^11^ It is difficult to interpret changes in tuberculosis testing given that the majority of laboratory registers did not record a referral source in this category.

Referral sources were not reported for any tests by 46% (43/94) of laboratories in 2009 and 40% (40/101) of laboratories in 2011. It was not possible to infer the HIV status of these patients. In laboratories with unknown referrals amongst some tests, the intermittent lack of referral documentation may be due to random missing data at the laboratory. Alternatively, in some laboratories, technicians may prioritise documentation of samples from HIV clinics and leave all other referral sources blank, leading to samples referred from NHSs and those without a referral source being grouped together. In this scenario, patients with unknown referral sources would be more likely to represent the general population. If unknown referral sources were actually non-HIV patients, our data suggest little meaningful change in the proportion of tests referred from NHSs between 2009 and 2011, relative to tests referred by HIV services.

Limiting the analysis to laboratories that did record a referral source restricted the sample size to 51 in 2009 and 61 laboratories in 2011. Within this subgroup, the proportion of tests referred from NHSs increased across all test types, similar to analyses including the full sample of laboratories. A model was used to predict the odds that an individual test was referred from a NHS, given that individual tests are not independent of each other in a laboratory. Using this model, the probability of a test being referred from a NHS was two times higher in 2011 than in 2009. Notably, haematology and tuberculosis tests were less likely to be referred from a NHS in 2011 than in 2009. This finding may be due to the model taking into account the correlation of the laboratory tests within a specific site when estimating the odds ratios. For example, larger laboratories may have skewed the results reported in Figures 2 and 3, but once intrafacility correlation is accounted for, the proportion of tests referred from NHSs for haematology and tuberculosis appeared to decrease over time. These findings suggest a large amount of site-level variation in the odds of a test being referred from NHSs. In addition, the observed odds ratio for all tests was likely driven by the increases in NHS testing in chemistry and syphilis between 2009 and 2011.

### Limitations

This study has several limitations. Firstly, the data comprised a non-random convenience sample of laboratories. Thus, the results may not be generalisable to other PEPFAR-supported laboratories in Tanzania or in other PEPFAR-supported countries. It is also unknown, in the absence of a comparison group, whether the volume of laboratory tests referred from HIV services and NHSs would have changed in the absence of PEPFAR or at comparable public laboratories not supported by PEPFAR. Secondly, it would have been advantageous to describe the change in laboratory tests over a longer period. However, this was not feasible, as ICAP support for most study laboratories began in 2009. Thirdly, because the sources of the laboratory data did not record identifiable patient information, the unit of analysis in this study was a laboratory test and not an individual patient, who could have had multiple tests. As stated previously, the HIV status of the patient for whom each test was performed was unknown. Future analyses evaluating utilisation of laboratory services at the patient level would provide additional information as to whether there are differences in laboratory usage according to patients’ HIV status. Fourthly, our data did not include information on the reason for a test being ordered for samples referred from NHSs. Thus, we could draw no conclusions as to whether or how guidelines for laboratory testing amongst non-HIV patients influenced utilisation of laboratory services. Finally, we were limited by the availability of hard-copy laboratory registers. The absence of a register did not necessarily mean that tests were not performed in a given laboratory, but merely that we were unable to access documentation of the test being performed. Even when registers were available, only 54% (51/94) of laboratories in 2009 and 61% (61/101) of laboratories in 2011 recorded referral sources; amongst those that did, we could not verify the referral source against other records. However, data availability and quality are unlikely to have changed notably over the study period.

This study provides descriptive data as a departure point for answering the question of how PEPFAR’s investment in laboratory services may have influenced utilisation of laboratory services by the general population. A systematic impact evaluation would be beneficial and would require prospective data or comparison groups and should include data on other variables about serviced populations, including the HIV status of patients for whom laboratory tests are performed, and laboratory characteristics, including staffing, equipment, training, quality improvement and costs.

### Conclusion

This retrospective study found that in a convenience sample of PEPFAR-supported laboratories in Tanzania, the number and proportion of tests performed for patients referred from NHSs increased for all tests from 2009 to 2011 compared with referrals from HIV services. The increase was driven in part by chemistry and syphilis testing. Although these findings are descriptive and may not be generalisable to other HIV-supported laboratories in Tanzania and other resource-limited countries, this finding may reflect increased documentation of referrals from NHSs in laboratory registers over time. Another possibility is that laboratory scale-up originally intended to service the HIV-positive population in Tanzania may be associated with a ‘spillover effect’ on laboratory use amongst the general population in the sampled facilities. These data may inform subsequent prospective studies to evaluate the impact of PEPFAR-supported laboratory scale-up on utilisation of laboratory services and the impact on health outcomes amongst the general population
